# Correction: Clusters and associations of adverse neonatal events with adult risk of multimorbidity: A secondary analysis of birth cohort data

**DOI:** 10.1371/journal.pone.0330639

**Published:** 2025-08-20

**Authors:** Jeeva John, Seb Stannard, Simon D. S. Fraser, Ann Berrington, Nisreen A. Alwan

The authors, Jeeva John, Seb Stannard, Simon D. S. Fraser, Ann Berrington, Nisreen A. Alwan, should not have been attributed equal contribution to this work.

The authors, Jeeva John and Seb Stannard, should be noted as having contributed equally to this work.
