# Supplementary figures and images for: Clusters and associations of adverse neonatal events with adult risk of multimorbidity: A secondary analysis of birth cohort data
Source: PLoS One. 2025 Mar 18;20(3):e0319200. doi: 10.1371/journal.pone.0319200 (PMC11918344; doi:10.1371/journal.pone.0319200)

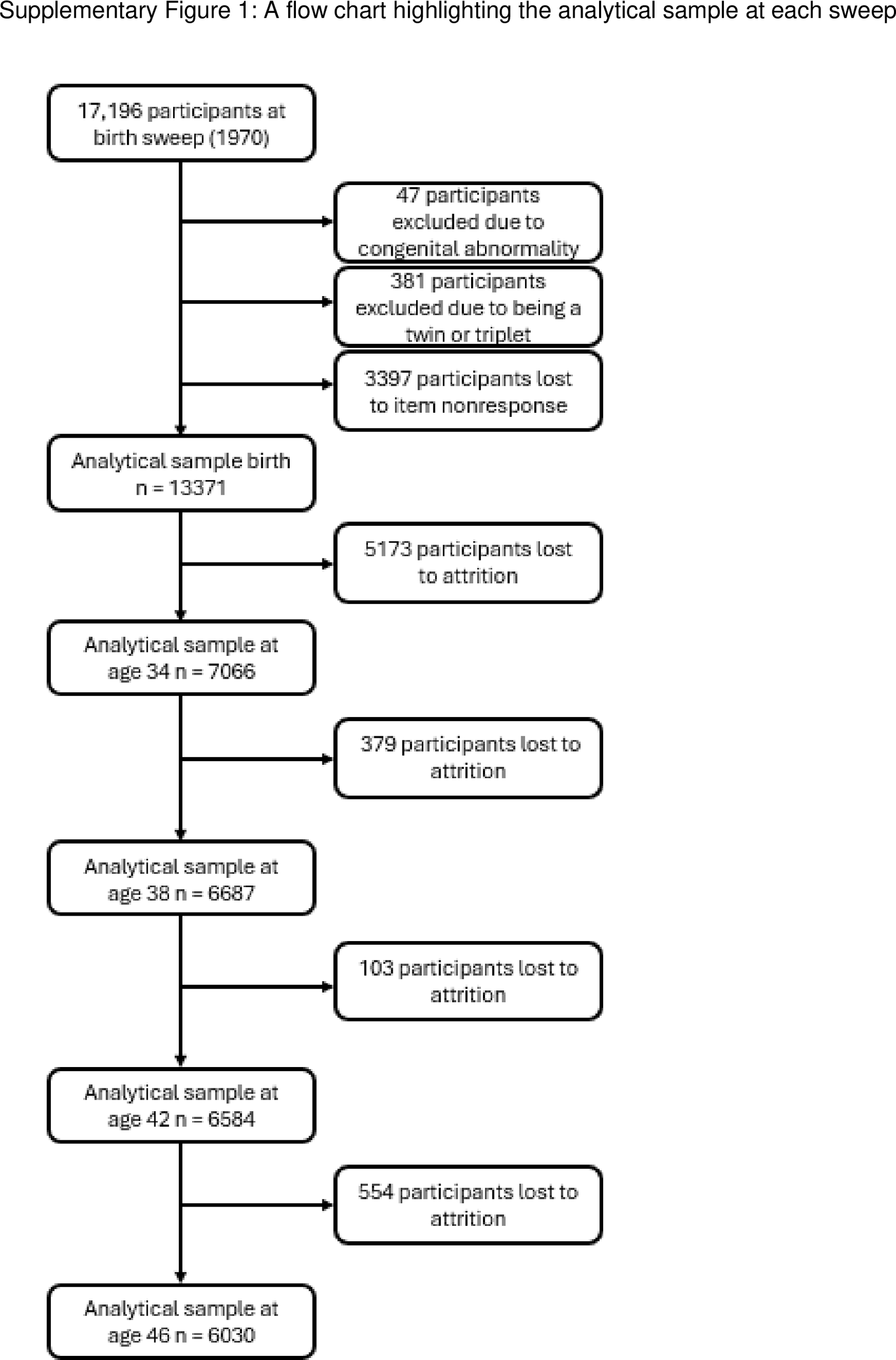

Supplement: S1 Fig — (TIF) [file pone.0319200.s001.tif]
