# Supplementary material for: Clusters and associations of adverse neonatal events with adult risk of multimorbidity: A secondary analysis of birth cohort data
Source: PLoS One. 2025 Mar 18;20(3):e0319200. doi: 10.1371/journal.pone.0319200 (PMC11918344; doi:10.1371/journal.pone.0319200)
Supplement: S1 Table — (DOCX) [file pone.0319200.s002.docx]

Supplementary Table 1: List of congenital abnormalities

*Hydrocephalus*

*Microcephalus*

*Spina bifida*

*Central nervous system malformations*

*Hiatus hernia*

*Tracheal/ oesophageal fistulae*

*Oesophageal atresia*

*Rectal/ anal atresia*

*Congenital heart disease*

*Cleft palate*

*Limb reduction deformities*

*Cystic hygroma*

*Exomphalos*

*Omphalocele*

*Chromosomal abnormalities*

*Other congenital abnormalities*
