# Supplementary material for: Clusters and associations of adverse neonatal events with adult risk of multimorbidity: A secondary analysis of birth cohort data
Source: PLoS One. 2025 Mar 18;20(3):e0319200. doi: 10.1371/journal.pone.0319200 (PMC11918344; doi:10.1371/journal.pone.0319200)
Supplement: S2 Table — (DOCX) [file pone.0319200.s003.docx]

Supplementary Table 2: Study variables

| **Neonatal variables (Birth Sweep) Value Score** | | |
| --- | --- | --- |
| *Gestational Age* | In days N/A  In grammes N/A | |
| *Birthweight* |  |  |
| *Duration to establish respiratory rate (Categories as per dataset)* | >3 minutes | 1 |
|  | <3 minutes | 0 |
| *Resuscitation* | Required any type of resuscitation. | 1 |
|  | No resuscitation required. | 0 |
| *Cyanosis* | Present | 1 |
|  | Absent | 0 |
| *Cerebral signs*  *Included irritability, hypertonia, hypotonia, shrill cries, unspecified, hypocalcaemia* | Present | 1 |
|  | Absent | 0 |
| *Cephalohaematoma* | Present | 1 |
|  | Absent | 0 |
| *Other illnesses*  *Included feeding difficulties, vomiting, failure to thrive, haemorrhages and Other(pyrexia, septicaemia, unspecified)* | Present | 1 |
|  | Absent | 0 |
| *Breathing difficulties*  *Included Respiratory Distress Syndrome (RDS), Intercostal rib recession, grunting/groaning, respiratory infection, apnoeic attacks, other* | Present | 1 |
|  | Absent | 0 |
