# Supplementary material for: Clusters and associations of adverse neonatal events with adult risk of multimorbidity: A secondary analysis of birth cohort data
Source: PLoS One. 2025 Mar 18;20(3):e0319200. doi: 10.1371/journal.pone.0319200 (PMC11918344; doi:10.1371/journal.pone.0319200)
Supplement: S3 Table — (DOCX) [file pone.0319200.s004.docx]

Supplementary Table 3: Structure of the first axis of the MCA conducted with 9 adverse neonatal indicators. *n* = 13,371 participants.

| Factor loading value | | | | | | |
| --- | --- | --- | --- | --- | --- | --- |
| *Continuous indicator* | *Range* | | *Mean(SD)* | | *Dim 1* | |
| **Birthweight(grammes)^a^**  GA(days) | | 567 – 6463 101 – 458 | | 3323.8*(520.1)*  281.6*(16.7)* | | -0.36  - |
| *Categorical indicator* | *Category* | | *n(%)* | | *Dim 1* | |
| **RR time(minutes)^a^**  Resuscitation  **Cyanosis^a^**  **Cerebral signs^a^**  Cephalohaematoma  **Other illnesses^a^**  **Breathing difficulties^a^** | >3  <3  Yes  No  Present  Absent  Present  Absent  Present  Absent  Present  Absent  Present  Absent | | 547(4.1)  549(4.1)  253(1.9)  216(1.6)  153(1.1)  432(3.2)  291(2.2) | | 0.60  -  0.88  0.69  -  0.36  0.90 | |

^a^Retained variables and factor loadings > 0.3 are in bold.

SD: Standard Deviation; GA: Gestational Age (in days); RR: Duration to establish Respiratory Rate
