# Supplementary material for: Clusters and associations of adverse neonatal events with adult risk of multimorbidity: A secondary analysis of birth cohort data
Source: PLoS One. 2025 Mar 18;20(3):e0319200. doi: 10.1371/journal.pone.0319200 (PMC11918344; doi:10.1371/journal.pone.0319200)
Supplement: S4 Table — (DOCX) [file pone.0319200.s005.docx]

Supplementary Table 4: Unadjusted association between multimorbidity and cohort member BMI and smoking status at ages 34, 38, 42 and 46.

| Age | | | | |
| --- | --- | --- | --- | --- |
| *n* 7,053 6,666 6,584 5,519 | | | | |
| BMI (kg/m^2^) | **34** | **38** | **42** | **46** |
| **18.5 - <25.0** | *Ref* | *Not available* | *Ref* | *Ref* |
| **<18.5** |  |  |  |  |
| Risk ratio (95% CI) | 0.93 (0.55 – 1.60) | - | 1.41 (0.96 – 2.08) | 1.14 (0.61 – 2.11) |
| *p* value | 0.80 |  | 0.08 | 0.68 |
| **25.0 - <30** |  |  |  |  |
| Risk ratio (95% CI)  *p* value | 1.01 (0.88 – 1.15)  0.91 | - | 1.11 (0.99 – 1.25)  0.07 | 1.12 (0.98 – 1.27)  0.09 |
| **>30**  Risk ratio (95% CI)  *p* value  Smoking status  **Never**  **Ex**  Risk ratio (95% CI)  *p* value  **Current**  Risk ratio (95% CI)  *p* value | 1.53 (1.33 – 1.77)  0.00*  *Ref*  1.15 (0.99 – 1.33)  0.07  1.45 (1.27 – 1.65)  0.00* | -  *Ref*  1.12 (0.97 – 1.29)  0.13  1.61 (1.41 – 1.84)  0.00* | 1.70 (1.52 – 1.90)  0.00*  *Ref*  1.21 (1.09 – 1.36)  0.00*  1.37 (1.22 – 1.63)  0.00* | 1.74 (1.55 – 1.96)  0.00*  *Ref*  1.26 (1.13 – 1.39)  0.00*  1.46 (1.31 – 1.64)  0.00* |
|  |  |  |  |  |

*Ref*: Reference group; *n* = number of participants included

*significant at <0.05 level
