# Supplementary material for: Clusters and associations of adverse neonatal events with adult risk of multimorbidity: A secondary analysis of birth cohort data
Source: PLoS One. 2025 Mar 18;20(3):e0319200. doi: 10.1371/journal.pone.0319200 (PMC11918344; doi:10.1371/journal.pone.0319200)
Supplement: Attachment — Submitted filename: Plos One response.docx [file pone.0319200.s008.docx]

The source of covariate information is still not available in the methods. Are they from self-reported questionnaires, clinical measurements during the study, medical records, registers, or somewhere else?

**Thank you. In line 185, we now state that the covariates were ‘recorded in self-reported questionnaires.’**

Statistical methods: Thank you for clarifying that the authors have used log-binomial regression instead of logistic regression. I would suggest that the authors do not mention logistic regression, but instead just mention log-binomial regression.

**Thank you. We have updated the manuscript from ‘logistic regression’ to ‘log-binomial regression’ throughout.**
